# Soil Conditions Rather Than Long-Term Exposure to Elevated CO_2_ Affect Soil Microbial Communities Associated with N-Cycling

**DOI:** 10.3389/fmicb.2017.01976

**Published:** 2017-10-18

**Authors:** Kristof Brenzinger, Katharina Kujala, Marcus A. Horn, Gerald Moser, Cécile Guillet, Claudia Kammann, Christoph Müller, Gesche Braker

**Affiliations:** ^1^Department of Biogeochemistry, Max Planck Institute for Terrestrial Microbiology, Marburg, Germany; ^2^Department of Plant Ecology, University of Giessen, Giessen, Germany; ^3^Water Resources and Environmental Engineering Research Unit, University of Oulu, Oulu, Finland; ^4^Department of Ecological Microbiology, University of Bayreuth, Bayreuth, Germany; ^5^Institute of Microbiology, Leibniz Universität Hannover, Hannover, Germany; ^6^Climate Change Research for Special Crops, Department of Soil Science and Plant Nutrition, Geisenheim University, Geisenheim, Germany; ^7^School of Biology and Environmental Science, University College Dublin, Dublin, Ireland; ^8^University of Kiel, Kiel, Germany

**Keywords:** elevated CO_2_, N_2_O, denitrifiers, ammonia oxidizers, N-fixers, DNRA, FACE

## Abstract

Continuously rising atmospheric CO_2_ concentrations may lead to an increased transfer of organic C from plants to the soil through rhizodeposition and may affect the interaction between the C- and N-cycle. For instance, fumigation of soils with elevated CO_2_ (*e*CO_2_) concentrations (20% higher compared to current atmospheric concentrations) at the Giessen Free-Air Carbon Dioxide Enrichment (GiFACE) sites resulted in a more than 2-fold increase of long-term N_2_O emissions and an increase in dissimilatory reduction of nitrate compared to ambient CO_2_ (*a*CO_2_). We hypothesized that the observed differences in soil functioning were based on differences in the abundance and composition of microbial communities in general and especially of those which are responsible for N-transformations in soil. We also expected *e*CO_2_ effects on soil parameters, such as on nitrate as previously reported. To explore the impact of long-term *e*CO_2_ on soil microbial communities, we applied a molecular approach (qPCR, T-RFLP, and 454 pyrosequencing). Microbial groups were analyzed in soil of three sets of two FACE plots (three replicate samples from each plot), which were fumigated with *e*CO_2_ and *a*CO_2_, respectively. N-fixers, denitrifiers, archaeal and bacterial ammonia oxidizers, and dissimilatory nitrate reducers producing ammonia were targeted by analysis of functional marker genes, and the overall archaeal community by 16S rRNA genes. Remarkably, soil parameters as well as the abundance and composition of microbial communities in the top soil under *e*CO_2_ differed only slightly from soil under *a*CO_2_. Wherever differences in microbial community abundance and composition were detected, they were not linked to CO_2_ level but rather determined by differences in soil parameters (e.g., soil moisture content) due to the localization of the GiFACE sets in the experimental field. We concluded that +20% *e*CO_2_ had little to no effect on the overall microbial community involved in N-cycling in the soil but that spatial heterogeneity over extended periods had shaped microbial communities at particular sites in the field. Hence, microbial community composition and abundance alone cannot explain the functional differences leading to higher N_2_O emissions under *e*CO_2_ and future studies should aim at exploring the active members of the soil microbial community.

## Introduction

Due to anthropogenic emissions, atmospheric CO_2_ concentrations are rising by about 1% per year and are expected to double in this century (IPCC, 2014) causing well known-climatic effects. Observations from the world-wide longest lasting CO_2_ enrichment study, the Giessen Free Air Carbon Dioxide Enrichment (GiFACE since 1998, ongoing), showed that elevated atmospheric CO_2_ (*e*CO_2_) concentrations also exert several impacts on soil communities. For instance, plant biomass was stimulated by ~12–15% (Kammann et al., 2005; Andresen et al., 2017). It is thus hypothesized that an increased transfer of organic C from plants to the soil through rhizodeposition occurs which affects soil microbial communities with implications for the interaction between C- and N-cycling (Freeman et al., 2004; Denef et al., 2007). A meta-analysis of greenhouse gas emission data from CO_2_ enrichment experiments demonstrated that increased CO_2_ generally stimulated emissions of nitrous oxide (N_2_O), another potent greenhouse gas, from terrestrial ecosystems (van Groenigen et al., 2011). At GiFACE for instance, long-term N_2_O emissions under *e*CO_2_ increased more than 2-fold compared to ambient CO_2_ (*a*CO_2_), but the underlying mechanisms are not fully resolved yet (Kammann et al., 2008). In two ^15^N tracing laboratory experiments with soils from FACE sites in Giessen and New Zealand gross N-transformations under *e*CO_2_ shifted toward a higher importance of heterotrophic processes (Müller et al., 2009; Rütting et al., 2010). In addition, turnover of ammonia (heterotrophic nitrification) and the rates of dissimilatory reduction of nitrate to ammonia (DNRA) increased, while turnover of nitrate was reduced. At GiFACE, ammonia concentrations under *e*CO_2_ were on average 17% higher while nitrate concentrations were significantly lower than at ambient CO_2_ (Müller et al., 2009).

Changes in gross N-transformations and gaseous N emissions are dependent on the dynamics and activity of microbial communities. In soils, N_2_O is mainly produced by denitrifiers and nitrifiers (Conrad, 1996; Butterbach-Bahl et al., 2013) and alterations in the functioning of denitrifiers and ammonia oxidizers in soils exposed to *e*CO_2_ were clearly discernable (e.g., Barnard et al., 2005, 2006). However, little information is available to date on how these functional shifts may be related to shifts in the underlying microbial communities and the understanding of potential feedback effects resulting in higher N_2_O emissions is still limited. Several studies found profound differences in abundance and composition between the overall microbial communities in soils exposed to elevated and ambient CO_2_ (Denef et al., 2007; Drigo et al., 2008, 2009; He et al., 2010; Deng et al., 2012; Xu et al., 2013; Dunbar et al., 2014; Xiong et al., 2015; Xia et al., 2017). Elevated levels of CO_2_ were also reported to influence microbial communities associated with N-cycling. Two early cultivation based studies showed an enhanced abundance of nitrate dissimilating *Pseudomonas* in the rhizosphere of grasses at *e*CO_2_ (Fromin et al., 2005; Roussel-Delif et al., 2005). Lesaulnier et al. (2008) found a significant decrease of nitrate reducers and crenarchaeal ammonia oxidizers with *e*CO_2_ and field exposure of a grassland ecosystem to *e*CO_2_ for 10 years significantly increased the abundance of N-fixers and *nirS*-type denitrifiers (He et al., 2010). Interestingly, in two out of three replicate FACE plots studied at GiFACE, the ratio of N_2_O reducers to nitrite reducers was lower under *e*CO_2_ (Regan et al., 2011) and may thus explain higher N_2_O fluxes from the soil (Philippot et al., 2011). In a California grassland, the structure and abundance of the ammonia oxidizing bacterial community was altered by *e*CO_2_, strongly interacting with the factor precipitation (Horz et al., 2004). Horz et al. (2004) also showed that multifactorial global change (*e*CO_2_, temperature, precipitation, N-deposition) fed back into the enrichment of a specific clade of ammonia oxidizers related to *Nitrosospira* spp. with higher potential for nitrification.

Alteration of microbial communities associated with soil functioning such as N-cycling suggests concomitant alterations of potential functional activity and hence of ecosystem functioning (He et al., 2010). We hypothesized that the increased N_2_O emissions in response to *e*CO_2_ (Kammann et al., 2008) and shifts in N-transformations observed at Giessen FACE during long-term exposure to elevated CO_2_ levels (Müller et al., 2009; Rütting et al., 2010) lead to differences in soil parameters (e.g., soil nitrate) and can be explained by changes of microbial communities in general and particularly of communities associated with N-cycling. To explore the microbial communities, we applied a molecular approach to study the abundance and composition based on functional marker genes for denitrification (*nirK*/*nirS, nosZ*), ammonia oxidation (bacterial and archaeal *amoA*), nitrogen fixation (*nifH*), dissimilatory nitrate reduction to ammonia (DNRA, *nrfA*) as well as archaeal and bacterial communities (16S rRNA genes). We used different molecular techniques (qPCR, T-RFLP, and 454 pyrosequencing), of which each satisfies specific demands in microbial community analyses. In addition to bias introduced by the use of gene specific primers, each technique, however, has its limitations but their complementary results have the potential to provide more comprehensive insights. Screening techniques (qPCR and T-RFLP) allow for a comparative assessment of microbial community abundance and composition in high numbers of replicate samples. An appropriate level of replication is a prerequisite for downstream statistical analyses of the data sets. While these approaches do not provide information on the identity of organisms, thousands of sequences generated through 454 pyrosequencing on the other hand, allow separating genotypes of functional marker genes and thus provide an in depth analysis of community composition.

## Materials and methods

### Site description and sampling

Soil samples were taken from the GiFACE experiment site (50°32′N and 8°43.3′E; 172 m a.s.l.) near Giessen, Germany. Within the GiFACE experiment CO_2_ fumigation on an old grassland site (>100 years) was started in May 1998 to study the response of a semi-natural grassland to long-term, moderate atmospheric CO_2_ enrichment of 20% above ambient (Jäger et al., 2003). The whole facility consists of six circular plots, each with 8 m internal diameter. Two plots build one set each (numbered 1, 2, and 3) with an ambient (*a*CO_2_, labeled with A) and an elevated (*e*CO_2_, labeled with E) CO_2_ plot. In 1998, the ambient CO_2_ concentration in the *a*CO_2_ plots was 364 and 399 ppm in *e*CO_2_. In 2012, when this study was conducted, the CO_2_ concentrations were 390 and 422 ppm, respectively. The three sets of plots are located on a gradual terrain slope (2°) in the direction of the rivulet Lückebach, which causes a gradient in soil moisture during spring and summer, and therefore drier conditions in set 1 compared to the others. The evapotranspiration during the plant growth period differed between *a*CO_2_ and *e*CO_2_ plots and resulted in soil moisture differences (Figure S2). The soil in the GiFACE plots was classified as a Fluvic Gleysol and has a sandy clay loam texture on top of a clay layer that varies in depth between the three sets (Jäger et al., 2003). The soil was characterized by a mean C and N content of 4.5 and 0.45%, respectively, and had a pH of ~6.2. Mean annual precipitation was 550 mm and mean annual air temperature was 9.6°C during the observation period from 1996 to 2003. Vegetation is the same in all plots and is dominated by 12 grass species, 2 legumes, and 15 non-leguminous herbs, and is characterized for all six plots as an *Arrhenatheretum elatioris* Br. Bl. *Filipendula ulmaria* subcommunity. The grassland has not been plowed for at least 100 years. It has been managed for several decades as a hay meadow with two cuts per year, and fertilized in mid-April with granular mineral calcium-ammonium-nitrate fertilizer at the rate of 40 kg N ha^−1^ yr^−1^ since 1996; before 1996, it was fertilized at 50–100 kg N ha^−1^ yr^−1^ (Kammann et al., 2008). The aboveground plant biomass harvest in *e*CO_2_ plots was in most years significantly higher (8–16%) than in *a*CO_2_ plots, this CO_2_ fertilization effect was most prominent in years with average temperatures and soil moisture and disappeared in years with extreme climatic conditions (Obermeier et al., 2017).

In July 2012, three replicate soil core samples were taken inside each of the six plots at a depth of 0–7.5 cm at random locations east, south and west of the center. July was chosen for sampling because usually the most pronounced differences in soil functioning between ambient and elevated plots were found during the summer period. The samples (18 in total) were homogenized and divided into two equal portions. One portion of each sample was stored at −20°C until further molecular analyses in the laboratory and the other portion was stored at 4°C until soil analytics.

### Measurement of soil parameters

Soil parameters were regularly recorded since the start of the GiFACE facility in 1997. N_2_O flux, soil moisture content, and precipitation at the field site was measured as described by Kammann et al. (2008) and Regan et al. (2011). In brief, N_2_O fluxes were determined in triplicate in each GiFACE plot using chambers of 30 cm height (frustum shape; 0.184 m^3^ volume). Chambers were sealed for 60–90 min to permanently installed soil frames, and sampled four times in 20–30-min intervals (longer in winter-time where fluxes were lower) with 60-ml PE syringes, and N_2_O fluxes were calculated by linear regression. Samples were analyzed within 24 h after collection on a gas chromatograph (HP6890) equipped with an ECD. Part of the dataset (1997–2006, 2008) used in this study was published previously (Kammann et al., 2008; Regan et al., 2011), data for 2007 and from 2009 to 2013 were additionally included. Flux data as well as soil moisture content and precipitation for all plots were then outlined for these dates.

Soil pH, water content, nitrate (${\text{NO}}_{3}^{-}$)-, nitrite (${\text{NO}}_{2}^{-}$)-, and ammonia (${\text{NH}}_{4}^{+}$)-concentrations as well as total carbon (C) and nitrogen (N) content were determined from each soil core. Soil pH was determined after shaking a soil sample (10 g) in 25 mL CaCl_2_ solution (0.01 M CaCl_2_ × 2H_2_O; Merck, Germany) for 20 min followed by settling for 1 h in the dark at room temperature (Schinner et al., 1996). Suspension pH was measured with an InLab® semi-micro electrode (Mettler-Toledo GmbH, Giessen, Germany). Soil moisture content (%) was determined gravimetrically by drying 1 g of homogenized soil for 3 days at 65°C in a drying oven (Memmert GmbH & Co. KG, Schwabach, Germany). The moisture content was calculated from the sample weight before and after drying. Afterwards, dried samples were ground after addition of liquid nitrogen and aliquots were analyzed at the Chemical Department of the Phillips-University Marburg (Germany) with a CHN-elemental analyzer to determine the total C/H/N percentage concentration of the soil.

To measure ${\text{NO}}_{3}^{-}$, ${\text{NO}}_{2}^{-}$, and ${\text{NH}}_{4}^{+}$ concentrations, 1 g of soil sample was suspended in 1 mL of Nuclease-free H_2_O and subsequently sterile-filtered with a disposable Filter Unit (0.2 μm; Whatman, MAGV, Germany). Concentrations of ${\text{NO}}_{3}^{-}$ and ${\text{NO}}_{2}^{-}$ were analyzed by ion chromatography (IC; Skyam GmbH Eresing, Germany; 70°C oven temperature) equipped with a LCA A14 column (Skyam GmbH, Eresing, Germany) using a 50 μL injection volume and Na_2_CO_3_ as eluent (flow of 1.5 mL min^−1^). The concentration of ${\text{NH}}_{4}^{+}$ in the soil samples was measured fluorometrically in triplicates by microscale analysis (Murase et al., 2006).

### Nucleic acid extraction

DNA was extracted from 0.35 g soil using the NucleoSpin® Soil Kit (Machery-Nagel GmbH & Co. KG, Düren, Germany) following the manufacturer's protocol. Afterwards, the amount and purity of extracted DNA was determined with a NanDrop1000 Spectrophotometer (Thermo Scientific, Langenselbold, Germany). The concentration of DNA ranged from 100 to 120 ng μL^−1^. The ratios of A_260/280_ and A_260/230_ were 1.6–1.9 and 1.8–2.1, respectively, which indicated a high purity of the extracted DNA with minimum contamination of e.g., proteins and phenol (A_260/280_) and carbohydrates (A_260/230)_.

### Quantification of functional marker and 16S rRNA genes

Copy numbers of genes encoding the denitrification associated enzymes nitrite reductase (*nirK*/*nirS*) and nitrous oxide reductase (*nosZ)*, nitrogen fixation associated dinitrogenase (*nifH)*, nitrification associated archaeal and bacterial ammonia monooxygenase (*amoA)*, nitrite reductase associated with the dissimilatory reduction of nitrate to ammonia (*nrfA)* as well as archaeal and bacterial 16S rRNA were quantified by qPCR. Primers and PCR conditions used are given in Table S1. A typical reaction mixture contained 12.5 μL of SybrGreen Jump-Start ReadyMix (Sigma-Aldrich, Taufkirchen, Germany), 0.5 μM of each primer, 3–4.0 mM MgCl_2_, 2 μL of soil DNA except for amplification of *nosZ*, for which 3 μL of DNA were used. For the amplification of functional marker genes involved in nitrogen cycling 200 ng BSA mL^−1^ were added. All assays were performed in an iCycler (Applied Biosystems, Darmstadt, Germany). Standard curves were obtained using serial 10-fold dilutions of a known amount of plasmid DNA (10^8^ to 10^1^ gene copies) containing the respective gene fragment. Negative controls were always run with water instead of template DNA. PCR reactions were done with 1:50 and 1:100 diluted DNA extracts. Efficiencies for all assays were between 80 and 97% with *r*^2^-values between 0.971 and 0.996.

### Analysis of the composition of functional marker and 16S rRNA genes

The composition of microbial communities containing *nirK*/*nirS, nosZ*, archaeal and bacterial *amoA, nifH, nrfA*, and archaeal 16S rRNA genes was explored by terminal restriction length polymorphism (T-RFLP) and barcode labeled 454 pyrosequencing analyses of PCR amplified gene fragments. Overall bacterial community composition based on bacterial 16S rRNA genes was assessed by T-RFLP, pyrosequencing data are available from de Menezes et al. (2016). Primers and PCR conditions used are given in Table S2. The quantity and quality of PCR amplicons were analyzed by gel electrophoresis (1.5% w/v agarose) and staining gels with 3 × GelRed Nucleic Acid Stain (Biotium, Köln, Deutschland). PCR products of the expected size were excised from the gel and purified using the DNA Wizard® SV Gel-and-PCR-Clean-up system (Promega, Mannheim, Germany).

For T-RFLP, forward or reverse primers were 5′-6-carboxyfluorescein labeled and amplicons were hydrolyzed by the restriction enzymes (FastDigest, Fermentas, St. Leon-Rot, Germany) *HaeIII (nirK*/*nirS*), *HhaI* (*nosZ, nifH, nrfA*, and *amoA*) and *MspI* and *Taq1* (archaeal and bacterial 16SrRNA, respectively). Afterwards, reaction products were purified using the SigmaSpin™ Sequencing Reaction Clean-up Columns (Sigma-Aldrich) according to the manufacturer's instructions. Fluorescently labeled restriction fragments were separated on an ABI PRISM 3100 Genetic Analyzer (Applera Deutschland GmbH, Darmstadt, Germany). The lengths of fluorescently labeled terminal restriction fragments (T-RFs) were determined by comparison with an internal DNA fragment length standard (X-Rhodamine MapMarker® 30–1,000 bp; BioVentures, Murfreesboro, TN) using GeneMapper software (Applied Biosystems). Peaks with >1% of the total fluorescence of a sample and >30 bp length were analyzed by aligning fragments to the internal standard. Reproducibility of patterns was confirmed for repeated terminal restriction fragment length polymorphism (T-RFLP) analysis using the same DNA extracts of selected samples. A difference of <2 base pairs in estimated length between different profiles was the basis for considering fragments identical in size. Peak heights from different samples were normalized to identical total fluorescence units by an iterative normalization procedure (Dunbar et al., 2001).

For pyrosequencing, DNA extracts from the three replicate samples of each plot were pooled and PCR amplified using the primers used for T-RFLP but with barcode labels (6 bp) which were designed to differentiate between GiFACE plots (E1: ACACAC; E2: ATGTAT; E3: AGCAGC; A1: ATCATC; A2: AGACTA; A3: AGTCAT) and with annealing temperatures increased by 2°C due to barcode tagging (Table S2). DNA concentration was 90–180 ng as determined by a Qubit® 2.0 Fluorometer using the Quant-iT TM dsDNA BR Assay Kit (Invitrogen Darmstadt, Germany). Libraries were built by pooling amplicons (200 ng each) of each gene from soil of six GiFACE plots and subjected to barcode labeled 454 pyrosequencing (GATC, Köln, Germany).

### Sequence analysis

Sequence processing and analysis was done in Qiime 1.3 (qiime.org). Pyrosequencing and PCR errors of the reads were corrected using the AmpliconNoise pipeline (Quince et al., 2011). Sequences of functional marker genes (*nirK*/*nirS, nosZ*, archaeal and bacterial *amoA, nifH*, and *nrfA*) were clustered as described previously (Caporaso et al., 2010; Palmer and Horn, 2012) using threshold similarities of 92%, because this reflects the threshold value beyond which the number of OTUs stays stable (Palmer and Horn, 2012; Palmer et al., 2012). Archaeal 16S rRNA gene sequences were clustered at 97% threshold similarities. Representative sequences were determined for each OTU. For statistical comparison of gene diversity in the plots, alpha-diversity measures were calculated in Qiime from rarefied OTU tables as described elsewhere (Hughes and Hellmann, 2005; Palmer and Horn, 2012). Rarefied OTU tables were generated by randomly subsampling original OTU tables 100 times. A sampling depth of 400 sequences was chosen for archaeal and bacterial *amoA, nifH, nirK, nirS, nosZ*, and *nrfA* to allow comparison of diversity between the different functional marker genes, as the number of sequences obtained exceeded 400 for all genes and soils. Rarefied OTU tables of archaeal 16S rRNA gene sequences were generated at a sampling depth of 150 sequences. The low number of sequences, however, clustered in up to 23 OTUs and was therefore considered sufficient to represent the most abundant taxa. The raw sequences were deposited at NCBI under the biosample accession numbers SAMN07212133 (archaeal 16S rRNA), SAMN07212134 (archaeal *amoA*), SAMN07212135 (bacterial *amoA*), SAMN07212136 (*nifH*), SAMN07212137 (*nirK*), SAMN07212138 (*nirS*), SAMN07212139 (*nosZ*), and SAMN07212140 (*nrfA*). The sequences representing the OTUs for each functional marker gene were deposited at NCBI under the biosample accession numbers SAMN07276914 (archaeal 16S rRNA), SAMN07276915 (archaeal *amoA*), SAMN07276916 (bacterial *amoA*), SAMN07276917 (*nifH*), SAMN07276918 (*nirK*), SAMN07276919 (*nirS*), SAMN07276920 (*nosZ*), and SAMN07276921 (*nrfA*).

### Statistical analyses of collected data

All statistical analyses were done using the statistical software R (version 3.0.1, R Development Core Team, 2013). Significant differences in copy numbers of archaeal/bacterial 16S rRNA genes, archaeal and bacterial *amoA, nirK, nirS, nosZ, nifH*, and *nrfA* were assessed using ANOVA (*P* < 0.05). All quantitative data were log-transformed prior to analysis to satisfy the assumptions of homoscedasticity and normally distributed residuals.

The effect of soil parameters on T-RFLP community profiles was explored by canonical correspondence analysis (CCA). Statistical significance of the CCA was assessed using permutation test (1,000 iterations). All community composition data were log-transformed before analysis, in order to reach normal distribution.

## Results

### N_2_O flux and soil moisture content over a period of 12 years

**Figure 1:**
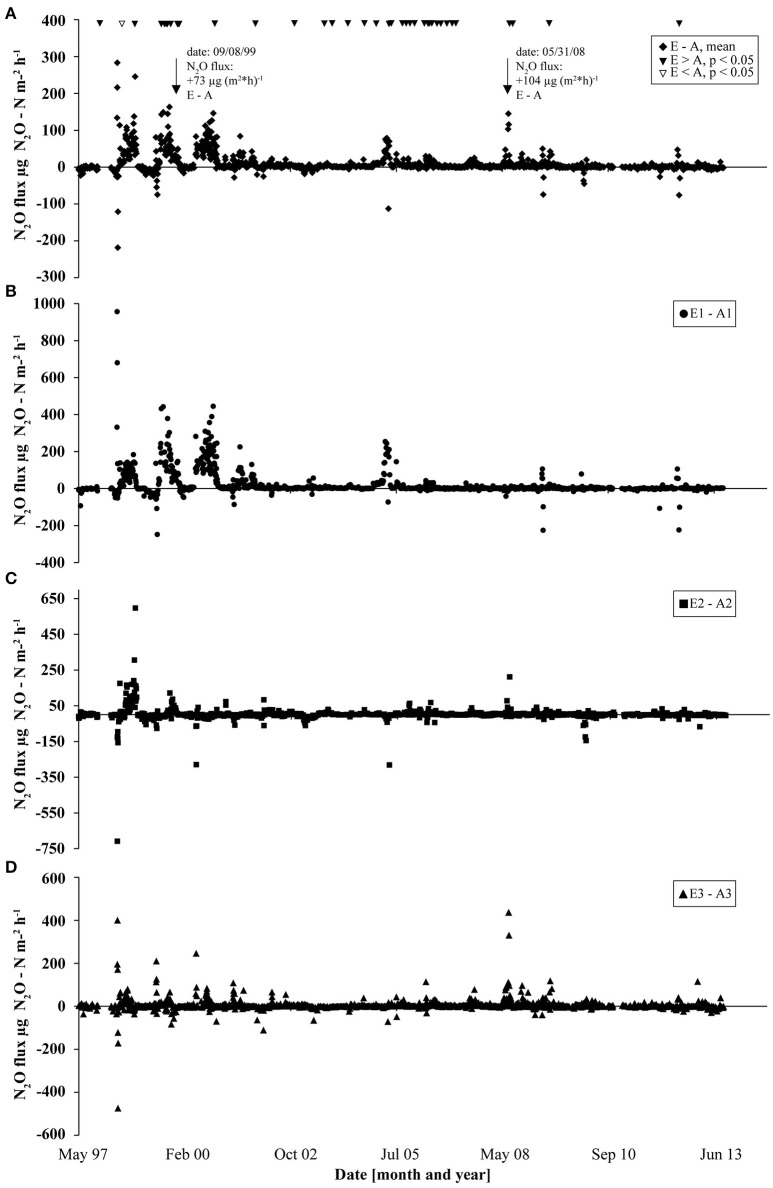
N_2_O flux measurements at GiFACE from 1997 to 2013 shown as the differences in fluxes between *e*CO_2_ and *a*CO_2_ plots. **(A)** Difference of mean N_2_O fluxes from soil at elevated (E) and ambient (A) CO_2_. Triangles mark occasions where N_2_O fluxes from *e*CO_2_ plots were significantly larger than from *a*CO_2_ plots (black triangles) or vice versa (white triangles) tested by ANOVA (*P* < 0.05). **(B–D)** Differences in N_2_O fluxes in the three sets (B, E1/A1; C, E2/A2; D, E3/A3).

N_2_O flux data collected since the start of the GiFACE facility in 1998 until June 2013 revealed an average flux of 21 μg N_2_O-N m^−2^ h^−1^ (1.84 kg N_2_O-N ha^−1^ yr^−1^) for the soil fumigated with *e*CO_2_ (E) and of 10 μg N_2_O-N m^−2^ h^−1^ (0.88 kg N_2_O-N ha^−1^ yr^−1^) for soil under *a*CO_2_ (A). The largest differences between fluxes from soil fumigated with *e*CO_2_ and *a*CO_2_ occurred during the first 3 years after the start of the experiment and the highest frequency of events occurred in years 2005–2006. Only one event occurred where the N_2_O flux from soil under *a*CO_2_ was higher (*P* < 0.05; Figure 1). Mostly but not generally, a rain event, which resulted in increased soil moisture content, preceded higher N_2_O fluxes (Figures S1, S2). For example, on day 866 (September 7, 1999) and on day 4055 (May 31, 2008) N_2_O fluxes from soil under *e*CO_2_ exceeded fluxes from soil under *a*CO_2_ by 73 and 104 μg N_2_O-N m^−2^ h^−1^, respectively. Both flux events were preceded by precipitation of 25.6 mm (day 865) and of 56.8 mm (day 4,054) which accounted for more than half of the cumulative precipitation in September 1999 (30.8 mm) and May 2008 (72.8 mm; Figure S1) which in consequence increased soil moisture concentration levels by more than 10% (Figure S2).

### Soil characteristics

Soil characteristics differed only marginally and non-significantly between plots fumigated with *e*CO_2_ and *a*CO_2_ (Table 1). Significant differences occurred only between GiFACE sets (Table S3) but not between *e*CO_2_ and *a*CO_2_ plots. The soil was moderately acidic with pH ranging from 5.45 to 6.10. Differences existed mostly between soil of the first set and the two other sets. In set E1/A1 pH (5.55) was lower than in set E2/A2 (6.03) and ${\text{NO}}_{3}^{-}$ concentration and C-content were lower than in E2/A2 and E3/A3, respectively. N-content was lower at E1/A1, while ${\text{NH}}_{4}^{+}$-concentration and C:N ratio was higher than in the two other sets. Water-content of the soil samples was similar in all plots.

**Table 1:** Characteristics of soil from GiFACE plots.

**Plot**	**Soil parameters**							
	**pH**	**${\text{NO}}_{3}^{-}$ [μM g^−1^ dw]**	**${\text{NH}}_{4}^{+}$ [μM g^−1^ dw]**	**H_2_O [%]**	**C [%]**	**H [%]**	**N [%]**	**C:N ratio**
E1	5.45^a^ ± 0.10	3.02^ab^ ± 0.32	0.37^a^ ± 0.076	25.00^a^ ± 2.00	4.50^a^ ± 0.24	0.98^ab^ ± 0.02	0.39^ab^ ± 0.02	11.43^a^ ± 0.10
A1	5.66^ab^ ± 0.30	2.14^a^ ± 0.99	0.30^ab^ ± 0.144	20.00^a^ ± 3.00	3.56^a^ ± 0.45	0.80^b^ ± 0.09	0.32^b^ ± 0.04	11.14^a^ ± 0.57
E2	6.04^b^ ± 0.12	8.02^b^ ± 3.75	0.23^ab^ ± 0.081	20.00^a^ ± 1.73	4.50^a^ ± 0.75	1.05^ab^ ± 0.09	0.44^ab^ ± 0.07	10.14^b^ ± 0.09
A2	6.02^b^ ± 0.16	4.71^ab^ ± 2.23	0.16^ab^ ± 0.002	22.67^a^ ± 0.58	4.56^a^ ± 0.83	1.03^ab^ ± 0.15	0.45^ab^ ± 0.07	10.04^b^ ± 0.25
E3	5.81^ab^ ± 0.27	3.77^ab^ ± 0.53	0.12^b^ ± 0.027	23.33^a^ ± 3.06	4.83^a^ ± 1.04	1.17^a^ ± 0.13	0.48^ab^ ± 0.09	10.10^b^ ± 0.19
A3	6.11^b^ ± 0.09	6.88^ab^ ± 1.40	0.20^ab^ ± 0.079	23.67^a^ ± 6.03	5.35^a^ ± 0.63	1.18^a^ ± 0.09	0.51^a^ ± 0.08	10.52^ab^ ± 0.69

ab *Identical letters indicate no significant differences (P > 0.05). Mean ± SD (n = 3)*.

### Abundance of microbial groups associated with soil nitrogen cycling

**Figure 2:**
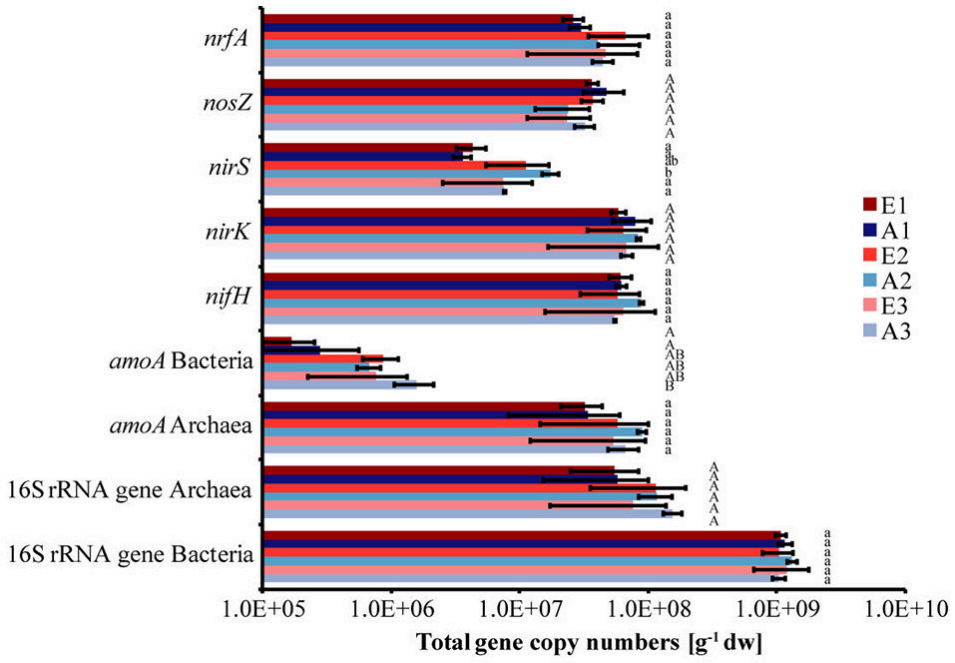
Abundance of dissimilatory nitrate reducers, denitrifiers, nitrogen fixers, ammonia oxidizers and total bacteria and archaea based on quantitative PCR analysis of the functional marker genes (*nrfA, nosZ, nirS, nirK, nifH*, archaeal and bacterial *amoA*) as well as of 16S rRNA genes, respectively. Bars indicate the total gene copy numbers. (Mean ± SD, *n* = 3). Different letters indicate significant differences in the abundance of a functional group between plots.

Total bacterial 16S rRNA gene copy numbers were in the order of 1 × 10^9^ g^−1^ dw soil, while archaeal 16S rRNA gene copy numbers ranged between 5 × 10^7^ and 1 × 10^8^ g^−1^ dw soil for all plots (Figure 2). The abundance of bacteria and archaea did not differ significantly between plots or sets (Figure 2). The absolute copy numbers of the functional marker genes *nirK, nosZ, nrfA, nifH*, and archaeal *amoA* (Figure 2) and their numbers relative to total (bacterial + archaeal) 16S rRNA gene copies were similarly high in all plots (Table 2, Figure S2). The genes *nirS* and bacterial *amoA* were 5- to 10-fold and 100-fold less abundant than the other functional marker genes, respectively (Figure 2). Absolute and relative copy numbers of *nirS* in plot A2 were similar to numbers in plot E2 but significantly higher than in each individual plot of the sets E1/A1 and E3/A3 (Figure 2, Table S4). Relative numbers were also significantly higher in set E2/A2 than in the other two sets (Table 2). Absolute and relative numbers of *bacterial amoA* in plot A3 were significantly higher than in plots A1 and E1 (Figure 2, Table S4) and relative numbers of bacterial *amoA* in set E3/A3 were significantly higher than in set E1/A1 (Table 2). Comparison of archaeal *amoA* and 16S rRNA gene copy numbers indicated that a large fraction of archaea harbored a copy of the *amoA* gene (ratios close to one, data not shown). For denitrification genes, copy numbers of *nirK* exceeded *nirS* which is also reflected in higher ratios of *nosZ*/*nirS* of 2–13 compared to 0.36–0.58 for *nosZ*/*nirK* which was low in all plot (Figure S3).

**Table 2:** Abundance of functional marker genes (archaeal and bacterial *amoA, nirK, nirS, nosZ, nrfA*, and *nifH*) relative to total 16S rRNA gene abundance (archaeal + bacterial) in soil of GiFACE sets E1/A1, E2/A3 and E3/A3.

	**Ratio (copy number of functional marker gene/total 16S rRNA genes)**						
**Set**	**Denitrification**			**Nitrification**		**DNRA**	**N-fixation**
	***nirK***	***nirS***	***nosZ***	**Archaeal *amoA***	**Bacterial *amoA***	***nrfA***	***nifH***
E1/A1	0.058^a^ ± 0.012	0.003^a^ ± 0.001	0.035^a^ ± 0.01	0.028^a^ ± 0.015	0.0002^a^ ± 0.0002	0.024^a^ ± 0.004	0.052^a^ ± 0.006
E2/A2	0.057^a^ ± 0.014	0.010^b^ ± 0.002	0.028^a^ ± 0.011	0.054^a^ ± 0.018	0.0007^ab^ ± 0.0004	0.042^a^ ±0.026	0.055^a^ ± 0.011
E3/A3	0.052^a^ ± 0.017	0.006^a^ ± 0.002	0.022^a^ ± 0.006	0.048^a^ ± 0.021	0.0010^b^ ± 0.0005	0.034^a^ ± 0.013	0.044^a^ ± 0.016

ab *Identical letters indicate no significant differences (P > 0.05). Mean ± SD (n = 6)*.

### Composition of microbial communities involved in soil nitrogen cycling

Applying a threshold similarity of 92% (97% for archaeal 16S rRNA genes) to sequences obtained from pyrosequencing, coverage of the libraries was in the range of 77.2–100% (Table 3). For *amoA* (archaeal and bacterial) the number of operational taxonomic units (OTUs) was low with only 3–7 OTUs observed and 3-8 OTUs estimated. Hence diversity calculated from rarefied tables (Shannon Diversity index H_arch. *amoA*_ = 0.90–1.59; H_bact. *amoA*_ = 0.64–1.37) was also low. Evenness of the archaeal ammonia oxidizer community and of the overall archaeal communities ranged from 0.47 to 0.64 and from 0.65–0.73, respectively, as the communities were dominated by only two *amoA* (OTU 1, 51.5–71.1%; OTU 2, 26.6–35.4%) as well as by one 16S rRNA (OTU 1, 34.7–42.0%) genotypes in all GiFACE plots (Table S5). These genotypes were closely related to *amoA* and the 16S rRNA gene from Candidatus *Nitrosphaera gargensis* and Cand. *Nitrosphaera viennensis*, respectively. Evenness (E = 0.25–0.50) was even lower for bacterial ammonia oxidizers which were dominated by two *amoA* genotypes (OTU 1, 75.4–84.2%; OTU 2, 14.2–22.5%) closely related to *amoA* of *Nitrospira* spp.

**Table 3:** Analysis of representative, Qiime-clustered sequences of PCR amplified gene fragments from GiFACE soil.

**Gene marker**	**Threshold similarity (%)**	**FACE ring**	**No. of sequences^a^**	**Good's coverage (%)^a^^,^^c^**	**No. of OTUs observed^b^**	**No. of OTUs estimated^d^**	**H^b^^,^^e^**	**E^b^^,^^f^**
*nifH*	92	E1	521	93.9	66	106	4.84	0.80
		A1	576	89.6	80	208	5.05	0.80
		E2	756	92.5	83	164	5.07	0.80
		A2	733	91.8	85	179	5.25	0.82
		E3	431	92.8	61	122	4.53	0.76
		A3	778	93.2	67	153	4.55	0.75
*nirK*	92	E1	881	94.3	108	178	4.82	0.71
		A1	1196	94.8	99	180	4.11	0.62
		E2	1275	95.5	98	176	4.21	0.63
		A2	904	94.5	93	178	4.39	0.67
		E3	2075	95.8	103	215	4.61	0.69
		A3	2370	96.3	108	210	4.31	0.64
*nirS*	92	E1	607	96.7	46	80	2.96	0.54
		A1	1373	97.6	45	84	2.80	0.51
		E2	2004	97.5	50	117	3.65	0.65
		A2	1324	97.3	53	108	3.75	0.65
		E3	1909	98.4	43	92	2.81	0.52
		A3	2384	98.2	52	106	3.60	0.63
*nosZ*	92	E1	432	92.4	57	107	3.40	0.58
		A1	840	95.4	44	100	2.90	0.53
		E2	1247	95.3	70	137	4.64	0.76
		A2	1073	95.1	62	128	4.27	0.72
		E3	1196	95.6	57	121	3.96	0.68
		A3	1510	95.3	74	151	4.50	0.72
*nrfA*	92	E1	1147	77.2	182	559	6.53	0.87
		A1	4999	86.1	177	596	6.35	0.85
		E2	5015	88.5	154	570	5.85	0.80
		A2	4778	87.3	154	590	5.78	0.80
		E3	6928	98.8	19	60	1.72	0.41
		A3	6583	95.0	60	233	2.80	0.47
Archaeal	92	E1	586	99.7	7	8	1.59	0.58
*amoA*		A1	2269	100.0	5	5	1.31	0.58
		E2	2137	100.0	4	4	0.85	0.47
		A2	565	100.0	3	3	1.02	0.64
		E3	2966	99.9	4	4	0.90	0.52
		A3	3547	100.0	4	4	1.08	0.54
Bacterial	92	E1	1119	99.9	4	4	0.79	0.42
*amoA*		A1	1099	100.0	7	7	1.37	0.50
		E2	2170	100.0	5	6	0.93	0.43
		A2	2839	100.0	4	5	0.75	0.38
		E3	1044	99.9	5	6	0.80	0.35
		A3	815	99.6	6	8	0.64	0.25
Archaeal	97	E1	827	98.0	15	25	2.61	0.68
16S rRNA		A1	2241	99.0	14	21	2.47	0.65
gene		E2	201	93.2	21	42	2.99	0.68
		A2	191	92.5	23	54	3.26	0.72
		E3	249	95.0	21	35	2.98	0.68
		A3	320	97.0	19	29	3.09	0.73

a *Numbers are based on original sequence data sets*.

b *Numbers are based on rarefied sequence data sets*.

c *Percent library coverage (Good's coverage): C = (1 − ns/nt) × 100, where ns is the number of OTUs that occur only once and nt is the total number of sequences*.

d *Chao 1 richness*.

e *Shannon diversity index*.

f *Species evenness*.

Numbers of observed OTUs for marker genes for N-fixation, denitrification and DNRA were at least one order of magnitude higher than for archaeal and bacterial *amoA* genes (Table 3). Communities of N-fixers, denitrifiers, and dissimilatory nitrate reducers were also more diverse than ammonia oxidizers (H_*nifH*_ = 4.53–5.25; H_*nirK*_ = 4.11; H_*nirS*_ = 2.80–3.75; H_*nosZ*_ = 2.90–4.64; H_*nrfA*_ = 1.72–6.53) and also more even (E_*nifH*_ = 0.76–0.82; E_*nirK*_ = 0.62–0.71; E_*nirS*_ = 0.51–0.65; E_*nosZ*_ = 0.53–0.76; E_*nrfA*_ = 0.41–0.87). The lowest evenness levels of E = 0.41 and 0.47 were found for DNRA communities of plot E3 and A3.

Generally, OTUs were most closely related to genes originating from as yet uncultured species but sequence identities of >71% to genes from cultivated species known to be involved in N-cycling confirmed that these genes were indeed derived from organisms of the respective target group. OTUs representing species of *Bradyrhizobium* were most abundant among N-fixers and nitrite reducers, while an OTU representing *Rhodopseudomonas palustris* dominated the *nosZ*-containing denitrifier communities. Communities of organisms capable of DNRA in sets E1/A1 and E2/A2 were not dominated by single OTUs and sequences were most closely related to *nrfA* from *Bacteroides* spp., *Anaromyxobacter* spp., *Sorangium* spp., and *Geobacter* spp.

### Influence of soil characteristics on the composition of microbial communities involved in soil nitrogen cycling

**Figure 3:**
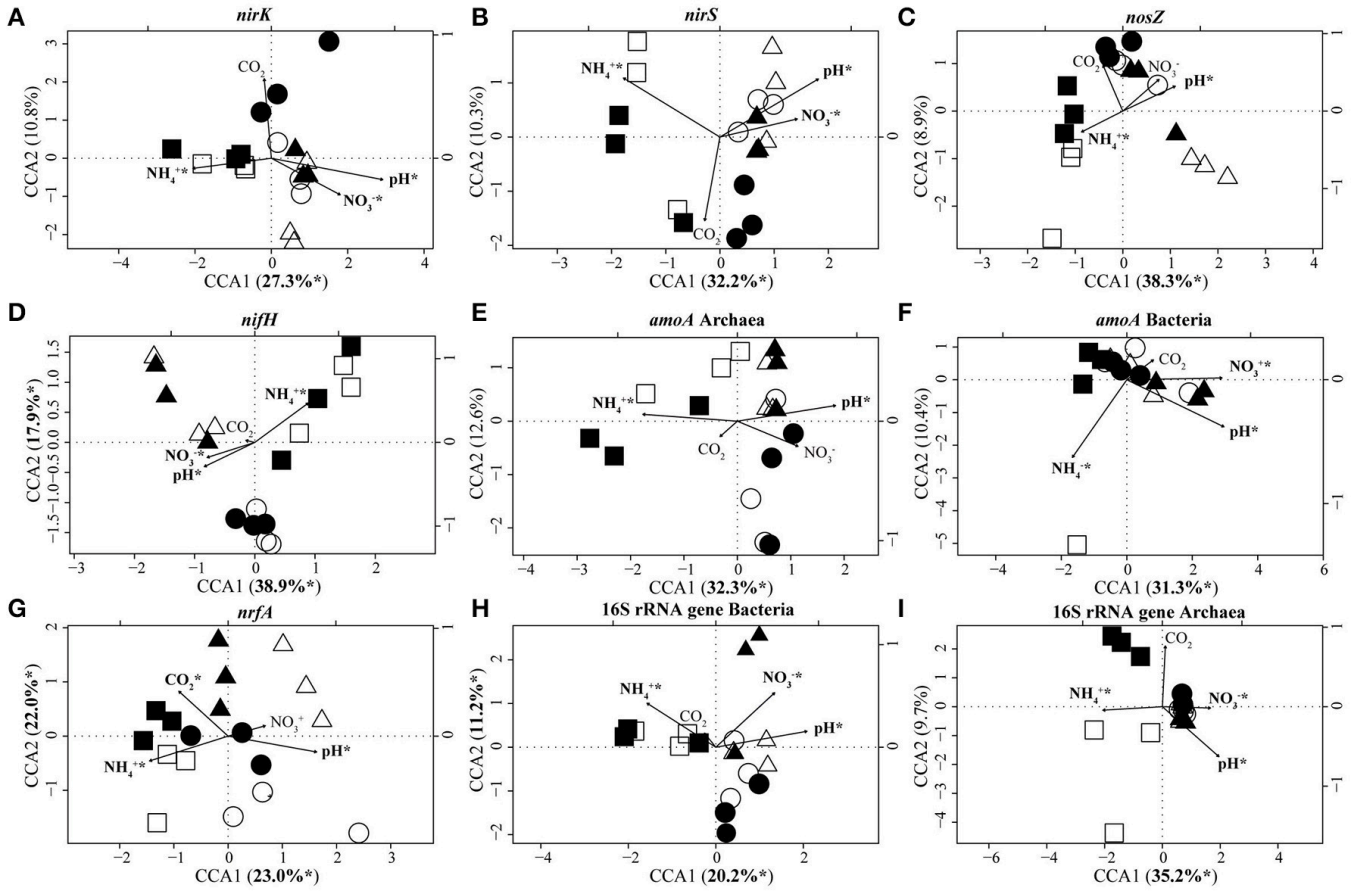
Canonical correspondence analysis (CCA) biplots based on T-RFLP community analyses of *nirK* **(A)**, *nirS* **(B)**, *nosZ* **(C)**, *nifH* **(D)**, archaeal *amoA* **(E)**, bacterial *amoA* **(F)**, *nrfA* **(G)**, bacterial 16S rRNA genes **(H)**, and archaeal 16S rRNA genes **(I)**. Arrows indicate the direction and relative importance (arrow lengths) of soil parameters associated with the clustering of the communities. For each gene the most important environmental variables are displayed and highlighted by an asterisk if significant in the model (ANOVA: *P* < 0.05). Square, triangle, and circle symbols represent sets E1/A1, E2/A2, and E3/A3, respectively. Closed symbols represent fumigation with *e*CO_2_ and open symbols the control plot at *a*CO_2_. (*n* = 3).

CCA based on T-RFLP data clustered N-fixer communities (*nifH*) into three distinct groups which corresponded to set E1/A1, E2/A2, and E3/A3 (Figure 3). Communities of denitrifiers (*nirK*/*nirS, nosZ*) and archaeal ammonia oxidizers (archaeal *amoA*) as well as the overall archaeal community (16S rRNA genes) in set E1/A1 were distinct from those in the other two sets. Lower pH and soil nitrate concentration as well as higher ammonia concentration separated communities of set E1/A1 from those of the other two sets (Table 1, Figure 3). The DNRA (*nrfA*), bacterial ammonia oxidizer (bacterial *amoA*) and overall bacterial communities (16S rRNA) showed no clustering according to GiFACE sets (Figure 3).

CCA identified pH (16.4–29.6% of the variance; Table 4) and ammonia concentration (12.9–30.7% of the variance; Table 4) as the most important soil parameters to shape the soil microbial communities. Both exerted a significant impact on microbial community composition independent of the gene considered. Nitrate concentration (5.2–20.3% of the variance; Table 4) also determined the composition of the microbial communities except for the communities of *nosZ*-containing denitrifiers, dissimilatory nitrate reducers, and archaeal ammonia oxidizers. The level of CO_2_, whether elevated or ambient, influenced the composition of dissimilatory nitrate reducer communities but the other communities were unaffected (Table 4).

**Table 4:** Proportion of variance in soil microbial communities in soil of GiFACE plots explained by environmental variables (percentage of total variation).

**Community**	**Variable**	**% Variance explained**	***P*-value**
*nifH*	pH value	17.3	**0.010**
	${\text{NO}}_{3}^{-}$ concentration	14.5	**0.023**
	${\text{NH}}_{4}^{+}$ concentration	20.6	**0.010**
	Elevated or ambient CO_2_	4.6	0.632
*nirK*	pH value	23.0	**0.005**
	${\text{NO}}_{3}^{-}$ concentration	11.5	**0.031**
	${\text{NH}}_{4}^{+}$ concentration	14.0	**0.015**
	Elevated or ambient CO_2_	6.8	0.231
*nirS*	pH value	21.2	**0.005**
	${\text{NO}}_{3}^{-}$ concentration	13.0	**0.030**
	${\text{NH}}_{4}^{+}$ concentration	20.1	**0.015**
	Elevated or ambient CO_2_	6.1	0.354
*nosZ*	pH value	20.3	**0.010**
	${\text{NO}}_{3}^{-}$ concentration	9.5	0.093
	${\text{NH}}_{4}^{+}$ concentration	12.9	**0.046**
	Elevated or ambient CO_2_	6.3	0.372
*nrfA*	pH value	16.4	**0.010**
	${\text{NO}}_{3}^{-}$ concentration	5.2	0.539
	${\text{NH}}_{4}^{+}$ concentration	14.5	**0.017**
	Elevated or ambient CO_2_	12.7	**0.020**
Archaeal *amoA*	pH value	24.5	**0.005**
	${\text{NO}}_{3}^{-}$ concentration	11.9	0.056
	${\text{NH}}_{4}^{+}$ concentration	22.5	**0.005**
	Elevated or ambient CO_2_	3.9	0.816
Bacterial *amoA*	pH value	26.7	**0.005**
	${\text{NO}}_{3}^{-}$ concentration	16.8	**0.018**
	${\text{NH}}_{4}^{+}$ concentration	18.3	**0.007**
	Elevated or ambient CO_2_	5.9	0.424
Bacterial 16S rRNA gene	pH value	19.4	**0.005**
	${\text{NO}}_{3}^{-}$ concentration	12.5	**0.017**
	${\text{NH}}_{4}^{+}$ concentration	13.5	**0.010**
	Elevated or ambient CO_2_	6.4	0.343
Archaeal 16S rRNA gene	pH value	29.7	**0.005**
	${\text{NO}}_{3}^{-}$ concentration	20.3	**0.017**
	${\text{NH}}_{4}^{+}$ concentration	30.7	**0.005**
	Elevated or ambient CO_2_	10.2	0.145

*CCA was applied to T-RFLP data of PCR amplified gene fragments of functional marker genes of the nitrogen cycle (nifH, nirK, nirS, nosZ, nrfA, archaeal and bacterial amoA) and of archaeal and bacterial 16S rRNA genes*.

*Bold numbers indicate significant differences between the sets tested by ANOVA (P value < 0.05)*.

Exploring whether CO_2_ exerted an influence on community composition in single sets showed that different microbial communities were affected. The level of CO_2_ determined the composition of the archaeal community in set E1/A1, the bacterial community and *nirS*-type denitrifiers in set E2/A2, and of dissimilatory nitrate reducers in set E3/A3 (Table 5).

**Table 5:** Influence of elevated atmospheric CO_2_ on the composition of microbial communities associated with nitrogen cycling in soil of GiFACE sets E1/A1, E2/A2, and E3/A3.

**Marker gene**	**GiFACE set**		
	**E1/A1**	**E2/A2**	**E3/A3**
*nirK*	0.700	0.401	0.197
*nirS*	0.201	**0.033**	0.082
*nosZ*	0.193	0.100	0.401
*nifH*	0.087	0.100	0.600
*nrfA*	0.151	0.125	**0.010**
Archaeal *amoA*	0.600	0.801	0.401
Bacterial *amoA*	0.418	0.084	0.056
Bacterial 16S rRNA gene	0.533	**0.001**	0.100
Archaeal 16S rRNA gene	**0.043**	0.415	0.053

*CCA was applied to T-RFLP data based on PCR amplified gene fragments of functional marker genes (nifH, nirK, nirS, nosZ, nrfA, archaeal and bacterial amoA) and of archaeal and bacterial 16S rRNA genes*.

*Bold numbers indicate significant differences between the sets tested by ANOVA (P < 0.05)*.

## Discussion

Given the functional differences in N-cycling observed by FACE experiments in Giessen and around the world (Kammann et al., 2008; Müller et al., 2009; Rütting et al., 2010; van Groenigen et al., 2011), we hypothesized adaptation of the soil microbial communities, i.e., differences in abundance and composition, to long-term exposure to elevated CO_2_. For the study period (1998–2013) we found 2-fold higher average N_2_O fluxes at *e*CO_2_. Thus, compared to the initial experimental period lasting from 1998 to 2006, the difference between fluxes at *e*CO_2_ and *a*CO_2_ (0.90 under *a*CO_2_ vs. 2.07 kg N_2_O–N ha^−1^ y^−1^ under *e*CO_2_) remained stable (Kammann et al., 2008). The continuous difference in functionality, altered N transformation rates as well as lower nitrate and higher ammonia concentrations observed at the GiFACE under elevated CO_2_ (Müller et al., 2009) suggest differences in the underlying microbial communities and particularly of communities associated with N-cycling. Our study, however, shows that soil microbial communities were surprisingly unaffected by elevated levels of CO_2_. In contrast to our hypothesis, the abundance and composition of the soil microbial communities associated with N-cycling in a given GiFACE set (E1 vs. A1, A2 vs. E2 and E3 vs. A3) were largely unaffected by CO_2_ level. A lack of response of microbial communities against long-term exposure to *e*CO_2_ was reported previously (Haase et al., 2008; Nelson et al., 2010; Marhan et al., 2011; Regan et al., 2011; Pujol Pereira et al., 2013; Dunbar et al., 2014). In addition, de Menezes et al. (2016) showed for GiFACE that *e*CO_2_ exerted no substantial effects on the composition of the overall soil bacterial community. Likewise, microbial communities involved in denitrification, ammonia oxidation, and DNRA remained unaffected by *e*CO_2_ in other studies (Deiglmayr et al., 2004; Haase et al., 2008; Marhan et al., 2011; Pujol Pereira et al., 2013).

These results, however, disagree with other studies that showed effects of *e*CO_2_ on soil microbial communities in general (Drigo et al., 2008, 2009; He et al., 2010; Deng et al., 2012; Xu et al., 2013; Dunbar et al., 2014; Xiong et al., 2015; Xia et al., 2017) or on communities associated with N-cycling (Horz et al., 2004; Fromin et al., 2005; Roussel-Delif et al., 2005; Lesaulnier et al., 2008; He et al., 2010; Regan et al., 2011). Yet, a comparison of results from different FACE facilities is ambiguous as the CO_2_ concentration applied (up to an overall concentration of +50%) varied. Denef et al. (2007) for instance, demonstrated even for GiFACE differences in PFLA patterns between soil fumigated with *e*CO2 and *a*CO_2_ albeit at concentrations of +50%. Changes in community composition and abundance were also observed in rice root samples, but again in response to higher elevation of CO_2_ (Okubo et al., 2015). Higher *e*CO_2_ levels may have resulted in a higher C-input into the soil by plants in these studies even though the total amount of nitrogen is almost equal between the study sites (Feng et al., 2015). Depending on the plant population the amount of C provided by the plants differs between 20 and 50% of total CO_2_ uptake (Kuzyakov and Domanski, 2000) and only a small fraction is available for microbial biomass production (van Veen et al., 1991). Thus, it remains unclear whether an elevation of CO_2_ by +20% suffices to increase C-inputs into the rhizosphere and raises the question whether a threshold in CO_2_ concentration may exist where not only the activity of the soil microbial communities is affected but also a community response will be detectable.

Haase et al. (2008) attributed the lack of response in microbial community abundance to unaltered C-flux from the whole root system of *Phaseolus vulgaris* into the soil. At GiFACE, the additional C assimilation also did not result in increased soil C sequestration. Instead, a loss of soil C, together with the breakup of large macroaggregates, was detected and caused enhanced ecosystem respiration under *e*CO_2_ (Lenhart, 2008). Influence of higher labile C input by the plant-root system may occur only directly at the root-soil interface and would then be rapidly consumed by microorganisms attached or located around the roots (Haase et al., 2008). It was also reported that fungal biomass was more strongly influenced by elevated CO_2_ than bacterial biomass (Jones et al., 1998; Drigo et al., 2009), but other studies found a negligible effect on fungal communities by *e*CO_2_ (Guenet et al., 2012; Dunbar et al., 2014; Lee et al., 2015). In our study, the level of CO_2_ had a general effect on the composition of dissimilatory nitrate reducer communities and affected the composition of additional but distinct communities in one but not all GiFACE sets. The effect on dissimilatory nitrate reducers agrees well with the findings by Müller et al. (2009) that DNRA rates increased by ~150% while soil nitrate content decreased under *e*CO_2_.

The persisting differences in N_2_O fluxes (Kammann et al., 2008) suggested differences in soil parameters at elevated and ambient CO_2_ levels but neither lower soil nitrate levels under *e*CO_2_ occurred nor was any other soil parameter affected by *e*CO_2_. Presumably, the impact of cultivation as permanent grassland for over 100 years had a more profound effect on the soil characteristics than 14 years of moderate exposure to *e*CO_2_. In addition, the increase in atmospheric CO_2_ concentration from ~300 to 400 ppm in the last 100 years is larger than the experimental exposure at GiFACE. Hence, we assume that long-term cultivation and the increase in CO_2_ prior to the experimental period led to the development of microbial communities which are adapted to the prevailing soil conditions but seem unresponsive to moderately increased CO_2_ levels. The grassland soil has been under long-term stable management as a hay meadow and has not been plowed for at least 100 years (Kammann et al., 2008). Plowing, fertilization regime, and cropping with annual vs. perennial plants were shown to influence N_2_O fluxes and ammonia oxidizer and denitrifier soil bacterial communities (Thompson et al., 2016). Thompson et al. found decreased abundance and diversity of denitrifiers after plowing and ammonia oxidizer (archaeal and bacterial) communities which differed between soil cropped with annual and perennial plants.

In all plots a large fraction of sequences belonged to only a few OTUs which were most closely related to genes from a limited range of species. These groups may thus represent the well-adapted key players of N-cycling in the soil and occurred in almost identical relative abundance under *e*CO_2_ and *a*CO_2_. N-fixers and denitrifiers (*nirK, nirS*, and *nosZ*) were primarily represented by OTUs similar to functional genes of *Bradyrhizobia, a* group with known N-fixation and denitrification capability (Bedmar et al., 2005). Ammonia oxidizers were dominated by *Nitrosopira* spp., the prevalent bacterial ammonia oxidizer species in soils (Kowalchuk et al., 2000) and *Nitrososphaera* spp., a typical archaeal species in soils related to *Nitrosphaera viennensis* which was isolated from soil (Tourna et al., 2011; Stieglmeier et al., 2014). *Nitrososphaera* spp. was representative for both the archaeal ammonia oxidizer as well as the total archaeal community. Although bacterial and archaeal ammonia oxidizers clustered only into few OTUs, our results agree with previous findings on the relevance of these species in soils. OTUs representing *Bacteriodetes* and *Anaeromyxobacter* dominated the microbial community involved in DNRA. These species are known to also harbor a clade II *nosZ* gene (Sanford et al., 2012; Jones et al., 2013) which we did not study here but which may contribute to N_2_O metabolism at GiFACE.

Communities varied between different sets at GiFACE and were related to differences in soil parameters determined by the localization of the sets in the experimental field. A previous study found differences in the water level in the deeper soil layers at GiFACE in the order: E1/A1 < E3/A3 < E2/A2 (Lenhart, 2008). de Menezes et al. (2016) attributed differences in bacterial community composition at GiFACE to the soil moisture gradient. Long-term differences in the water level of the deeper soil layers probably determined the differences in pH and the concentration of C- and N-compounds observed in the top soil layer (0–7.5 cm) of the sets. Microbial community composition at GiFACE was influenced by pH, nitrate, and ammonia. Soil parameters are known as the predominant drivers determining the distribution of microorganisms and shaping their communities (e.g., Zhou et al., 2008). Variation in e.g., soil denitrifier (Enwall et al., 2010) and ammonia oxidizer communities (Wessén et al., 2010) was previously found to occur with spatial heterogeneity at scales similar to those at the GiFACE experimental site. Regan et al. (2011) also found stronger influence of the location of the GiFACE sets or the soil depth on the abundance of *amoA, nirK, nirS*, and *nosZ* than of *e*CO_2_. Likewise, Marhan et al. (2011) observed a similar trend and that temporal variation and soil depth had a greater effect on the abundance of nitrate reducers and bacteria than *e*CO_2_. Microbial communities in a soybean agroecosystem were also not affected by *e*CO_2_ but by lower soil moisture compared to ambient conditions (Pujol Pereira et al., 2013). Experimentally reduced precipitation increased the mass of microaggregates concomitant with a higher abundance of bacteria and *nosZ*-containing denitrifiers in microhabitats protected from reduced soil moisture (Pujol Pereira et al., 2013).

## Conclusion

The long-term cultivation as grassland as well as the increase in atmospheric CO_2_ in the past 100 years seems to have shaped soil microbial communities which density and composition remained unaltered by moderately *e*CO_2_. Despite a lack of response of microbial communities associated with N-cycling, N-transformations were altered and average N_2_O fluxes doubled under *e*CO_2_. Large fluxes were related to a high N-status of the soil after fertilization and plant growth in spring. This correlation suggests differential response to altered environmental conditions against the background of a community which remained unaltered. Palmer et al. (2016), for instance, observed differential transcriptional activation of denitrifiers in response to drying-rewetting and flooding. Hence, future studies should explore in more detail how elevated CO_2_ in conjunction with massive N inputs during fertilization impact microbial communities in the soil and whether this leads to a short-term activation of microbial groups involved in N-cycling and hence higher production of N_2_O.

## Author contributions

KB designed the study, performed the field sampling, performed all lab work (nucleic-acid extractions, T-RFLP analysis, qPCR analysis, preparation of 454 pyrosequencing), performed statistical analysis, evaluated the data and wrote the manuscript. KK performed the analysis of 454 pyrosequencing data and revised the manuscript. MH performed the analysis of 454 pyrosequencing data and revised the manuscript. GM designed the study, provided climate data and revised the manuscript. CK provided climate and N_2_O data and revised the manuscript. CG designed the gas analyses study and performed sampling, provided N_2_O data and revised the manuscript. CM developed the research idea, designed the study and revised the manuscript. GB developed the research idea, designed the research, and wrote the manuscript.

### Conflict of interest statement

The authors declare that the research was conducted in the absence of any commercial or financial relationships that could be construed as a potential conflict of interest.
