# Supplementary material for: Soil Conditions Rather Than Long-Term Exposure to Elevated CO2 Affect Soil Microbial Communities Associated with N-Cycling
Source: Front Microbiol. 2017 Oct 18;8:1976. doi: 10.3389/fmicb.2017.01976 (PMC5651278; doi:10.3389/fmicb.2017.01976)
Supplement: Supplementary file 1 [file Table1.pdf]

Table S1. Primers and PCR conditions used to amplify fragments of functional marker genes *nirK*, *nirS*, *nosZ*, *nifH*, archaeal and bacterial *amoA*, *nrfA*, and archaeal and bacterial 16S rRNA genes by qPCR.

Gene	Primer sets	Forward primer	Reverse primer	PCR conditions	PCR product length (bp)	References
nifH	PolF/ PolR	TGCGA(C/T)CC(G/C)A ARGC(C/G/T)GACTC	AT(G/C)GCCATCAT(C/T) TC(A/G)CCGGA	95 °C/15min, 6 cycles of (95°C/15sec, 60°C/30sec (-1°C every cycle), 72°C/30sec, 80°C/15sec), 40 cycles (95°C/15sec, 55°C/15sec, 72°C/30sec, 80°C/15sec), 60 to 95°C (+0.2°C/sec) for denaturation curve.	360	Poly *et al*., 2001
nirK	qnirK876/ qnirK1040	AT(C/T)GGCGG(A/C/G) A(C/T)GGCGA	GCCTCGATCAG(A/G)TT (A/G)TGGTT	95 °C/15min, 6 cycles of (95°C/15sec, 63°C/30sec (-1°C every cycle), 72°C/30sec, 80°C/15sec), 40 cycles (95°C/15sec, 58°C/30sec, 72°C/30sec, 80°C/15sec), 60 to 95°C (+0.2°C/sec) for denaturation curve.	165	Henry *et al*., 2004
nirS	qCd3af/ qR3cd	AACG(C/T)(G/C)AAGG A(A/G)AC(G/C)GG	GA(G/C)TTCGG(A/G)TG (G/C)GTCTT(G/C)A(C/T)G AA	95 °C/15min, 6 cycles of (95°C/15sec, 63°C/30sec (-1°C every cycle), 72°C/30sec, 80°C/15sec), 40 cycles (95°C/15sec, 58°C/30sec, 72°C/30sec, 80°C/15sec), 60 to 95°C (+0.2°C/sec) for denaturation curve.	425	Kandeler *et al*., 2006
nosZ	nosZ2F/ nosZ2R	CGC(A/G)ACGGCAA (G/C)AAGGT(G/C) (A/C)(G/C)(G/C)GT	CA(G/T)(A/G)TGCA(G/T) (G/C)GC(A/G)TGGCAGA A	95 °C/15min, 6 cycles of (95°C/15sec, 65°C/30sec (-1°C every cycle), 72°C/30sec, 80°C/15sec), 40 cycles (95°C/15sec, 60°C/15sec, 72°C/30sec, 80°C/15sec), 60 to 95°C (+0.2°C/sec) for denaturation curve.	267	Henry *et al*., 2006
nrfA	nrfA2aw/ nrfAR1	CA(A/G)TG(C/T)CA (C/T)GT(C/G/T)GA (A/G)TA	T(A/T)(A/C/G/T)GGCAT (A/G)TG(A/G)CA(A/G)TC	95 °C/15min, 6 cycles of (95°C/15sec, 58°C/30sec (-1°C every cycle), 72°C/30sec, 80°C/15sec), 40 cycles (95°C/15sec, 53°C/15sec, 72°C/30sec, 80°C/15sec), 60 to 95°C (+0.2°C/sec) for denaturation curve.	269	Welsh *et al*., 2014; Mohan *et al*., 2004
archaeal *amoA*	Arch-amoAF/ Arch-amoAR	(G/C)TAATGGTCTGGC TTAGACG	GCGGCCATCCATCTGTA TGT	95 °C/15min, 6 cycles of (95°C/15sec, 58°C/30sec (-1°C every cycle), 72°C/30sec, 80°C/15sec), 40 cycles (95°C/15sec, 53°C/20sec, 72°C/30sec, 80°C/15sec), 60 to 95°C (+0.2°C/sec) for denaturation curve.	635	Francis *et al*., 2005
bacterial *amoA*	amoA-1F/ amoA2R	GGGGTTTCTACTGGT GGT	CCCCTC(G/T)G(G/C)AAA GCCTTCTTC	95 °C/15min, 6 cycles of (95°C/15sec, 65°C/30sec (-1°C every cycle), 72°C/30sec, 80°C/15sec), 40 cycles (95°C/15sec, 60°C/15sec, 72°C/30sec, 80°C/15sec), 60 to 95°C (+0.2°C/sec) for denaturation curve.	491	Rotthauwe *et al*., 1997
archaeal 16S rRNA gene	Ar364f/ Ar934br	CGGGG(C/T)GCA(G/C) CAGGCGCGAA	GTGCTCCCCCGCCAATT CCT	95 °C/15min, 6 cycles of (95°C/15sec, 56°C/30sec (-1°C every cycle), 72°C/30sec, 80°C/15sec), 40 cycles (95°C/15sec, 52°C/15sec, 72°C/30sec, 80°C/15sec), 60 to 95°C (+0.2°C/sec) for denaturation curve.	570	Burggraf *et al*., 1997; Großkopf *et al*., 1998
bacterial 16S rRNA gene	Ba519f/ Ba907r	CAGC(A/C)GCCGCGG TAA(A/C/G/T)(A/T)C	CCGTCAATTC(A/C)TTT (A/G)AGTT	95 °C/15min, 6 cycles of (95°C/15sec, 54°C/30sec (-1°C every cycle), 72°C/30sec, 80°C/15sec), 40 cycles (95°C/15sec, 49°C/15sec, 72°C/30sec, 80°C/15sec), 60 to 95°C (+0.2°C/sec) for denaturation curve.	388	Lane, 1991
