# Supplementary material for: Soil Conditions Rather Than Long-Term Exposure to Elevated CO2 Affect Soil Microbial Communities Associated with N-Cycling
Source: Front Microbiol. 2017 Oct 18;8:1976. doi: 10.3389/fmicb.2017.01976 (PMC5651278; doi:10.3389/fmicb.2017.01976)
Supplement: Supplementary file 2 [file Table2.pdf]

Table S2. Primers and PCR conditions used to PCR amplify fragments of the functional marker genes *nirK*, *nirS*, *nosZ*, *nifH*, archaeal and bacterial *amoA*, *nrfA*, and archaeal and bacterial 16S rRNA genes for T-RFLP and 454 pyrosequencing (without FAM label). For 454 pyrosequencing, the annealing temperature was increased by 2°C.

**Gene**	**Primer sets**	**Forward primer**	**Reverse primer**	**PCR conditions**	**PCR product length (bp)**	**References**
nifH	PolF-FAM/ PolR	TGCGA(C/T)CC(G/C) AARGC(C/G/T)GAC TC	AT(G/C)GCCATCAT (C/T)TC(A/G)CCGGA	95 °C 5min, 10 cycles of (95°C/30sec, 60°C/40sec (-0.5°C every cycle), 72°C/2min), 25 cycles (95°C/30sec, 55°C/40sec, 72°C/2min) 72°C/10min.	360	Poly *et al*., 2001
nirK	nirK1F/ nirK5R-FAM	GG(A/C)ATGGT (G/T)CC(C/G)TGGC A	GCCTCGATCAG(A/G) TT(A/G)TGG	95 °C/5min, 10 cycles of (95°C/30sec, 56°C/40sec (-0.5°C every cycle), 72°C/40sec), 25 cycles (95°C/30sec, 54°C/40sec, 72°C/40sec), 72°C/7min.	514	Braker *et al*., 1998
nirS	cd3aF-FAM/ R3cd	GT(C/G)AACGT (C/G)AAGGA(A/G)A C(C/G)GG	GA(C/G)TTCGG(A/G) TG(C/G)GTCTTG	95 °C/5min, 10 cycles of (95°C/30sec, 56°C/40sec (-0.5°C every cycle), 72°C/40sec), 25 cycles (95°C/30sec, 54°C/40sec, 72°C/40sec) 72°C/7min.	425	Throbäck *et al*., 2004
nosZ	NosF-FAM/ NosR	CG(C/T)TGTTC(A/C) TCGACAGCCAG	CATGTGCAG (A/C/G/T)GC(A/G)TG GCAGAA	95 °C 5min, 10 cycles of (95°C/30sec, 59°C/90sec (-0.5°C every cycle), 72°C/2min), 25 cycles (95°C/30sec, 56°C/40sec, 72°C/2min) 72°C/10min.	700	Klooset *al*., 2001
nrfA	nrfA2aw-FAM/ nrfAR1	CA(A/G)TG(C/T)CA (C/T)GT(C/G/T)GA (A/G)TA	T(A/T)(A/C/G/T)GGC AT(A/G)TG(A/G)CA (A/G)TC	95 °C 5min, 10 cycles of (95°C/30sec, 57°C/40sec (-0.5°C every cycle), 72°C/2min), 25 cycles (95°C/30sec, 53°C/30sec, 72°C/2min) 72°C/10min.	269	Welsh *et al*., 2014; Mohan *et al*., 2004
archaeal *amoA*	Arch-amoAF-FAM/ Arch-amoAR	(G/C)TAATGGTCTG GCTTAGACG	GCGGCCATCCATCT GTATGT	95 °C 5min, 10 cycles of (95°C/30sec, 57°C/90sec (-0.5°C every cycle), 72°C/2min), 25 cycles (95°C/30sec, 53°C/50sec, 72°C/2min) 72°C/10min.	635	Francis *et al*., 2005
bacterial *amoA*	amoA-1F-FAM/ amoA2R	GGGGTTTCTACTG GTGGT	CCCCTC(G/T)G(G/C) AAAGCCTTCTTC	95 °C 5min, 10 cycles of (95°C/30sec, 65°C/40sec (-0.5°C every cycle), 72°C/2min), 25 cycles (95°C/30sec, 60°C/40sec, 72°C/2min) 72°C/10min.	491	Rotthauwe *et al*., 1997
archaeal 16S rRNA gene	Ar109f/ Ar912r-FAM	AC(G/T)GCTCAGTA ACACGT	GTGCTCCCCCGCCA ATTCCT	95 °C 5min, 10 cycles of (95°C/30sec, 58°C/60sec (-0.5°C every cycle), 72°C/2min), 25 cycles (95°C/30sec, 53°C/60sec, 72°C/2min) 72°C/10min.	803	Großkopf *et al*., 1998; Lueders and Friedrich, 2000
bacterial 16S rRNA gene	Ba27f-FAM/ Ba907r	GAGTTTG((A/C)TCC TGGCTCAG	CCGTCAATTC(A/C)T TT(A/G)AGTT	95 °C 5min, 10 cycles of (95°C/30sec, 49°C/60sec (-0.5°C every cycle), 72°C/2min), 25 cycles (95°C/30sec, 44°C/30sec, 72°C/2min) 72°C/10min.	898	Weisburg *et al*., 1991; Lane, 1991
