# Supplementary material for: Soil Conditions Rather Than Long-Term Exposure to Elevated CO2 Affect Soil Microbial Communities Associated with N-Cycling
Source: Front Microbiol. 2017 Oct 18;8:1976. doi: 10.3389/fmicb.2017.01976 (PMC5651278; doi:10.3389/fmicb.2017.01976)
Supplement: Supplementary file 3 [file Table3.pdf]

Table S3. Characteristics of soil from GiFACE sets E1/A1, E2/A2, and E3/A3.

Set	Soil characteristics							
	pH	NO_3^- [μM g^{-1} dw]	NH_4^+ [μM g^{-1} dw]	H_2O [%]	C [%]	H [%]	N [%]	C:N ratio
E1/A1	5.55[a] ± 0.23	2.58[a] ± 0.81	0.33[a] ± 0.111	22.50[a] ± 3.56	4.03[a] ± 0.61	0.89[a] ± 0.11	0.35[a] ± 0.04	11.29[a] ± 0.40
E2/A2	6.03[b] ± 0.13	6.36[b] ± 3.30	0.19[b] ± 0.079	21.33[a] ± 1.86	4.53[ab] ± 0.71	1.04[ab] ± 0.11	0.45[b] ± 0.05	10.09[b] ± 0.18
E3/A3	5.96[ab] ± 0.24	5.33[ab] ± 1.95	0.16[b] ± 0.069	23.50[a] ± 4.28	5.09[b] ± 0.82	1.18[b] ± 0.10	0.49[b] ± 0.08	10.32[b] ± 0.51

[ab] Identical letters indicate no significant differences ($P > 0.05$). Mean ±SD (n=6).
