# Supplementary material for: Soil Conditions Rather Than Long-Term Exposure to Elevated CO2 Affect Soil Microbial Communities Associated with N-Cycling
Source: Front Microbiol. 2017 Oct 18;8:1976. doi: 10.3389/fmicb.2017.01976 (PMC5651278; doi:10.3389/fmicb.2017.01976)
Supplement: Supplementary file 4 [file Table4.pdf]

Table S4. Abundance of functional marker genes relative to total bacterial and archaeal abundance in soil of GiFACE plots.

Plot	Ratio (copy number of functional marker gene/total copy number of 16S rRNA genes)						
	Denitrification			Nitrification		DNRA	N-fixation
	nirK	***nirS***	***nosZ***	**archaeal *amoA***	**bacterial *amoA***	***nrfA***	***nifH***
E1	0.059[a] ± 0.014	0.004[a] ± 0.001	0.032[a] ± 0.002	0.029[a] ± 0.111	0.0002[a] ± 0.0001	0.023[a] ± 0.006	0.054[a] ± 0.008
A1	0.078[a] ± 0.010	0.003[a] ± 0.000	0.039[a] ± 0.016	0.028[a] ± 0.067	0.0003[a] ± 0.0003	0.024[a] ± 0.002	0.050[a] ±0.004
E2	0.071[a] ± 0.018	0.009[bc] ± 0.003	0.034[a] ± 0.012	0.047[a] ± 0.114	0.0009[ab] ± 0.0005	0.055[a] ±0.015	0.047[a] ±0.015
A2	0.058[a] ± 0.007	0.012[c] ± 0.003	0.021[a] ± 0.002	0.062[a] ± 0.332	0.0005[ab] ± 0.0001	0.028[a] ± 0.031	0.062[a] ± 0.005
E3	0.047[a] ± 0.022	0.005[ab] ± 0.003	0.018[a] ± 0.002	0.039[a] ± 0.112	0.0006[ab] ± 0.0004	0.031[a] ± 0.019	0.043[a] ± 0.027
A3	0.057[a] ±0.011	0.006[ab] ± 0.001	0.027[a] ± 0.005	0.056[a] ± 0.085	0.0013[b] ± 0.0004	0.037[a] ± 0.006	0.046[a] ± 0.006

[ab] Identical letters indicate no significant differences ($P > 0.05$). Mean±SD (n=3).
