# Supplementary material for: Soil Conditions Rather Than Long-Term Exposure to Elevated CO2 Affect Soil Microbial Communities Associated with N-Cycling
Source: Front Microbiol. 2017 Oct 18;8:1976. doi: 10.3389/fmicb.2017.01976 (PMC5651278; doi:10.3389/fmicb.2017.01976)
Supplement: Supplementary file 5 [file Table5.pdf]

Table S5. Operational Taxonomic Units (OTUs) representing microbial communities in soil of GiFACE sets E1/A1, E2/A2, and E3/A3. Sequences of PCR amplified fragments of functional marker genes of the nitrogen cycle (*nifH*, *nirK*, *nirS*, *nosZ*, archaeal and bacterial *amoA*, and *nrfA*) and of archaeal 16SrRNA genes were determined by pyrosequencing and most closely related sequences were revealed by BLAST search.

Gene	OTU (No.)	Relative abundance of OTUs per set (1/2/3) in %	Closest relative (accession No.)	Identity (%)	Closest cultured relative (accession No.)	Identity (%)
nifH	17	15.6/18.8/15.2	Uncult. bact. (KF847701)	92	*Bradyrhizobium denitrificans* LMG 8443 (AP012279)	88
	8	9.3/16.0/22.8	Uncult. bact. (HQ335832)	94	*Azospirillum brasilense* AWC8 (GQ161227)	86
	28	11.7/5.2/3.3	Uncult. bact. (KF847733)	99	*Halorhodospira halophila* DSM 244 (EF199951)	87
	7	1.6/6.1/9.8	Uncult. bact. (JX268406)	99	*Azospirillum brasilense* AWB4 (GQ161231)	89
	1	8.1/2.5/1.8	Uncult. bact. (KC667514)	99	*Mesorhizobium huakuii* (KF800056)	85
	3	7.8/2.4/1.7	*Azospirillum brasilense* Gr58 (FR745919)	90	*Azospirillum brasilense* Gr58 (FR745919)	90
	18	3.8/4.4/3.9	Uncult. bact. (JX865930)	90	*Desulfovibrio magneticus* RS-1 (AP010904)	84
	12	5.7/4.5/1.5	Uncult. soil bact. (DQ776436)	99	*Gluconacetobacter diazotrophicus* (AF030414)	90
	2	1.3/0.8/2.6	Uncult. bact. (AY601063)	93	*Methylobact.* sp. 4-46 (CP000943)	89
	6	1.0/1.9/3.6	Uncult. bact. (AY601063)	97	*Azospirillum brasilense* Sp245 (HE577327)	91
nirK	8	23.7/23.1/23.9	Uncult. bact. (DQ783977)	99	*Bradyrhizobium japonicum* USDA 6 (AP012206)	85
	27	19.1/19.0/16.7	Uncult. bact. (DQ783979)	99	*Bradyrhizobium* sp. ORS278 (CU234118)	85
	19	5.3/13.1/8.2	Uncult. bact. (DQ304355)	100	*Azospirillum lipoferum* A5 (HQ288913)	94
	3	10.2/3.7/7.5	Uncult. bact. (DQ784024)	100	*Bradyrhizobium japonicum* SEMIA 5079 (CP007569)	81
	2	7.3/3.7/5.2	Uncult. bact. (DQ783839)	100	*Mesorhizobium ciceri* WSM1271 (CP002448)	85
	34	1.8/1.2/6.1	Uncult. bact. (DQ783865)	100	*Rhodopseudomonas palustris* TIE-1 (CP001096)	86
	4	2.2/3.3/1.9	Uncult. bact. (DQ783332)	99	*Bradyrhizobium japonicum* SEMIA 5079 (CP007569)	89
	25	2.0/2.2/2.5	Uncult. bact. (EF645006)	100	*Bradyrhizobium* sp. GSM-471 (FN600571)	83
	66	1.1/2.3/2.8	Uncult. bact. (AY249359)	99	*Sinorhizobium fredii* HH103 (HE616890)	82
	79	0.5/3.5/2.1	Uncult. bact. (DQ783944)	96	*Rhodopseudomonas palustris* TIE-1 (CP001096)	84
nirS	2	53.8/32.6/42.3	Uncult. bact. (KC468992)	99	*Bradyrhizobium oligotrophicum* S58 (AP012603)	87
	6	14.1/13.9/11.3	Uncult. bact. (KC010976)	98	*Thiobacillus denitrificans* ATCC 25259 (CP000116)	80
	9	4.7/10.1/12.7	Uncult. bact. (AY583422)	95	*Rubrivivax gelatinosus* IL144 (AP012320)	79
	12	1.6/11.0/9.8	Uncult. bact. (HE818699)	88	*Azoarcus aromaticum* EbN1 (CR555306)	79
	3	0.7/3.3/1.9	Uncult. bact. (HE995561)	100	*Bradyrhizobium oligotrophicum* S58 (AP012603)	85
	4	3.0/0.7/0.7	Uncult. bact. (JN179277)	95	*Rhodanobacter* sp. D206a (AB480490)	92
	25	0.5/1.7/1.7	Uncult. bact. (KC010985)	94	*Thiobacillus denitrificans* ATCC 25259 (CP000116)	76
	80	0.3/2.5/0.8	Uncult. bact. (JN179307)	92	*Pseudomonas stutzeri* ATCC 17588 (CP002881)	76
	22	0.2/1.4/2.1	Uncult. bact. (GU393213)	94	*Rubrivivax gelatinosus* IL144 (AP012320)	80
	28	0.3/1.2/1.9	Uncult. bact. (EU650311)	94	*Azospirillum* sp. TSO28-1 (AB545704)	83

nosZ	1	58.5/38.1/42.6	Uncult. bact. (FN859706)	99	*Rhodopseudomonas palustris* HaA2 (CP000250)	91
	7	17.1/12.4/14.3	Uncult. bact. (FM993387)	99	*Bradyrhizobium* sp. GSM-467 (FN600633)	96
	8	2.2/5.6/4.8	Uncult. bact. (FN859751)	99	*Bradyrhizobium japonicum* USDA 110 (BA000040)	88
	2	0.9/3.4/5.3	Uncult. bact. (AY325632)	90	*Bradyrhizobium japonicum* USDA 110 (BA000040)	82
	5	2.8/3.4/2.2	Uncult. bact. (FN295856)	99	*Bradyrhizobiaceae* bact. D195a (AB480505)	96
	22	1.1/3.2/3.4	Uncult. bact. (FN859742)	99	*Rhodopseudomonas palustris* HaA2 (CP000250)	85
	23	0.4/3.8/2.7	Uncult. bact. (JQ038940)	93	*Oligotropha carboxidovorans* OM5 (CP002825)	83
	12	4.0/1.5/1.4	Uncult. bact. (FN295926)	94	*Azospirillum* sp. TSH10 (AB542250)	91
	3	1.2/2.4/2.3	Uncult. bact. (FN859774)	99	*Oligotropha carboxidovorans* OM5 (CP002825)	87
	28	0.4/3.8/2.7	Uncult. bact. (FN859905)	99	*Bradyrhizobium japonicum* USDA 110 (BA000040)	87
nrfA	22	2.4/14.7/54.0	*Bacteroides fragilis* 638R (FQ312004)	73	*Bacteroides fragilis* 638R (FQ312004)	73
	19	0.5/1.4/28.0	Uncult. bact. (JX293771)	75	*Anaeromyxobacter dehalogenans* 2CP-1 (CP001359)	74
	30	9.4/12.3/0.9	Uncult. bact. (JX293735)	77	*Anaeromyxobacter dehalogenans* 2CP-1 (CP001359)	73
	23	12.2/2.8/0.4	Uncult. bact. (JX293808)	88	*Sorangium cellulosum* So ce 56 (AM746676)	80
	41	0.5/7.3/0.7	Uncult. bact. (JX293737)	88	*Anaeromyxobacter dehalogenans* 2CP-1 (CP001359)	77
	48	2.5/5.2/0.8	*Geobacter* sp. M18 (CP002479)	71	*Geobacter* sp. M18 (CP002479)	71
	7	4.6/1.4/0.3	Uncult. bact. (JX293797)	88	*Anaeromyxobacter* sp. Fw109-5 (CP000769)	79
	6	4.8/0.6/0.7	Uncult. bact. (JX293771)	80	*Anaeromyxobacter* sp. Fw109-5 (CP000769)	72
	16	0.8/4.2/0.5	Uncult. bact. (JX293798)	89	*Sorangium cellulosum* So ce 56 (AM746676)	80
	277	0.5/2.3/0.3	Uncult. bact. (JX293810)	89	*Anaeromyxobacter dehalogenans* 2CP-1 (CP001359)	79
**archaeal *amoA***	2	51.5/71.7/68.4	Uncult. arch. (JQ750224)	100	Cand. *Nitrososphaera gargensis* Ga9.2 (CP002408)	80
	3	35.4/26.6/30.0	Uncult. bact. (KJ645270)	100	Cand. *Nitrososphaera gargensis* Ga9.2 (CP002408)	81
	1	11.1/0.0/0.1	Uncult. Thaumarchaeote (KC962900)	100	Cand. *Nitrososphaera gargensis* Ga9.2 (CP002408)	91
	6	0.2/1.5/1.4	Uncult. Crenarchaeote (JF748278)	100	Cand. *Nitrososphaera evergladensis* SR1 (CP007174)	80
	7	0.8/0.0/0.1	Uncult. arch. (JQ750204)	99	Cand. *Nitrososphaera evergladensis* SR1 (CP007174)	79
	4	0.9/0.0/0.0	Uncult. arch. (KF709843)	100	Cand. *Nitrososphaera gargensis* Ga9.2 (CP002408)	81
**bacterial *amoA***	2	75.4/84.2/76.7	Uncult. bact. (KC010733)	100	*Nitrosospira* sp. Wyke8 (EF175099)	99
	1	16.4/14.2/22.5	Uncult. ammonia-oxidizing bact. (HQ638973)	100	*Nitrosospira* sp. Nsp12 (AY123823)	97
	15	5.1/0.4/0.3	Uncult. ammonia-oxidizing bact. (KC454074)	99	*Nitrosospira* sp. Nsp65 (AY123839)	93
	6	2.0/0.6/0.2	Uncult. ammonia-oxidizing bact. (JF936483)	100	*Nitrosospira* sp. CT2F (AY189143)	99
	27	0.5/0.1/0.0	Uncult. bact. (KC010732)	100	*Nitrosolobus multiformis* (AF042171)	100
**archaeal 16S rRNA gene**	1	42.0/37.7/34.7	Uncult. thaumarchaeote (KF276537)	99	*Nitrososphaera viennensis* EN76 (CP007536)	94
	7	14.1/10.9/8.8	Uncult. archaeote (GQ127221)	99	*Nitrososphaera viennensis* EN76 (CP007536)	96
	4	16.8/5.3/2.7	Uncult. archaeote (AB848859)	99	*Nitrososphaera viennensis* EN76 (CP007536)	96
	13	3.8/11.2/17.3	Uncult. archaeote (JQ249749)	99	*Nitrososphaera viennensis* EN76 (CP007536)	96
	2	6.9/1.7/3.5	Uncult. archaeote (JN205391)	99	*Nitrososphaera viennensis* EN76 (CP007536)	98
	8	3.4/3.1/3.3	Uncult. archaeote (KU297805)	99	Cand. *Nitrosocosmicus exaquare* G61 (CP017922)	99
	12	3.1/5.7/13.2	Uncult. Thaumarchaeote (KF041033)	100	*Nitrososphaera viennensis* EN76 (CP007536)	96
	20	1.5/7.1/4.4	Uncult. thaumarchaeote (KF275858)	99	Cand. *Nitrosocosmicus exaquare* G61 (CP017922)	97
	54	0.7/0.0/0.5	Uncult. thaumarchaeote (KF275835)	99	*Nitrososphaera viennensis* EN76 (CP007536)	96
	10	0.8/2.4/1.7	Uncult. thaumarchaeote (KC505296)	97	*Nitrososphaera viennensis* EN76 (CP007536)	94
