# Supplementary figures and images for: Soil Conditions Rather Than Long-Term Exposure to Elevated CO2 Affect Soil Microbial Communities Associated with N-Cycling
Source: Front Microbiol. 2017 Oct 18;8:1976. doi: 10.3389/fmicb.2017.01976 (PMC5651278; doi:10.3389/fmicb.2017.01976)

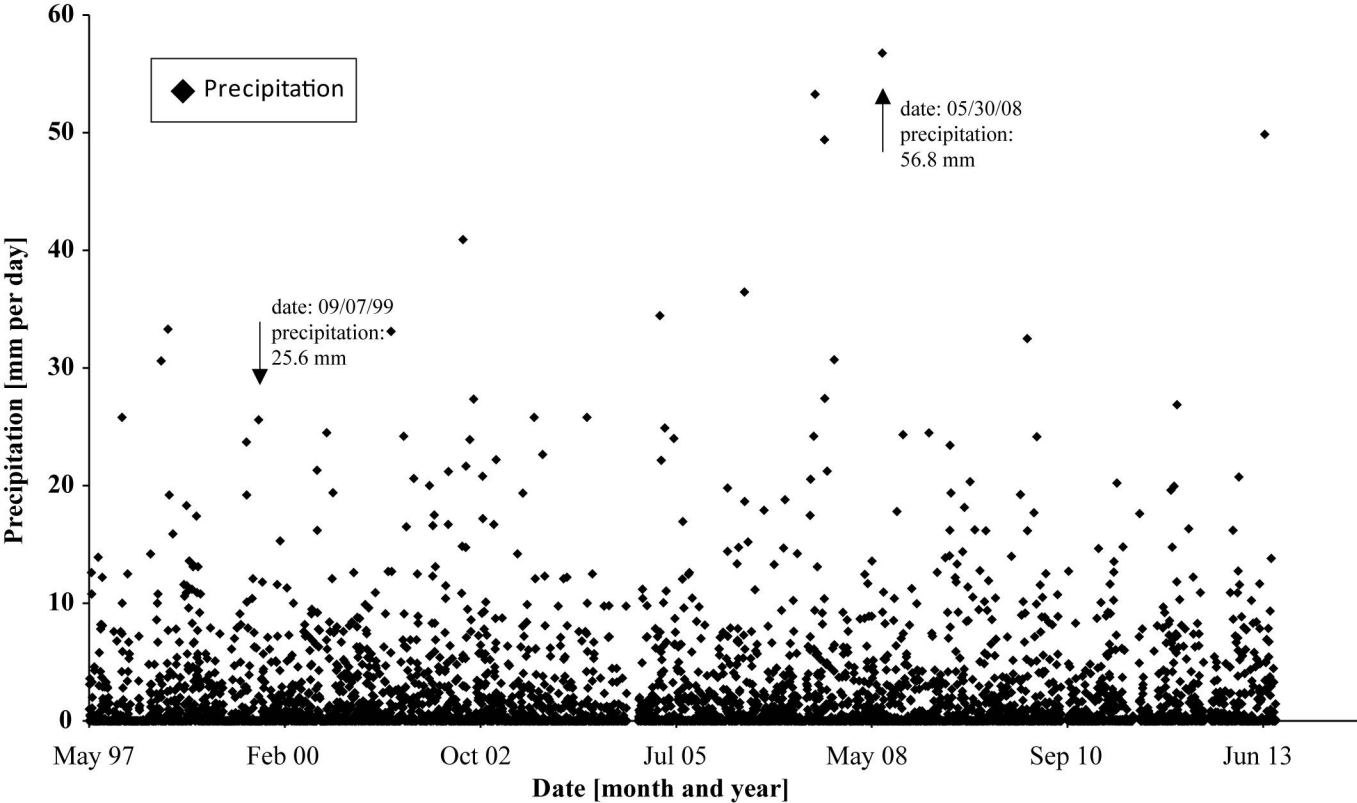

**Figure S1.** Precipitation at GiFACE from 1997–2013.

Supplement: Supplementary file 6 [file Image1.pdf]
