# Supplementary material for: Soil Conditions Rather Than Long-Term Exposure to Elevated CO2 Affect Soil Microbial Communities Associated with N-Cycling
Source: Front Microbiol. 2017 Oct 18;8:1976. doi: 10.3389/fmicb.2017.01976 (PMC5651278; doi:10.3389/fmicb.2017.01976)
Supplement: Supplementary file 7 [file Image2.pdf]

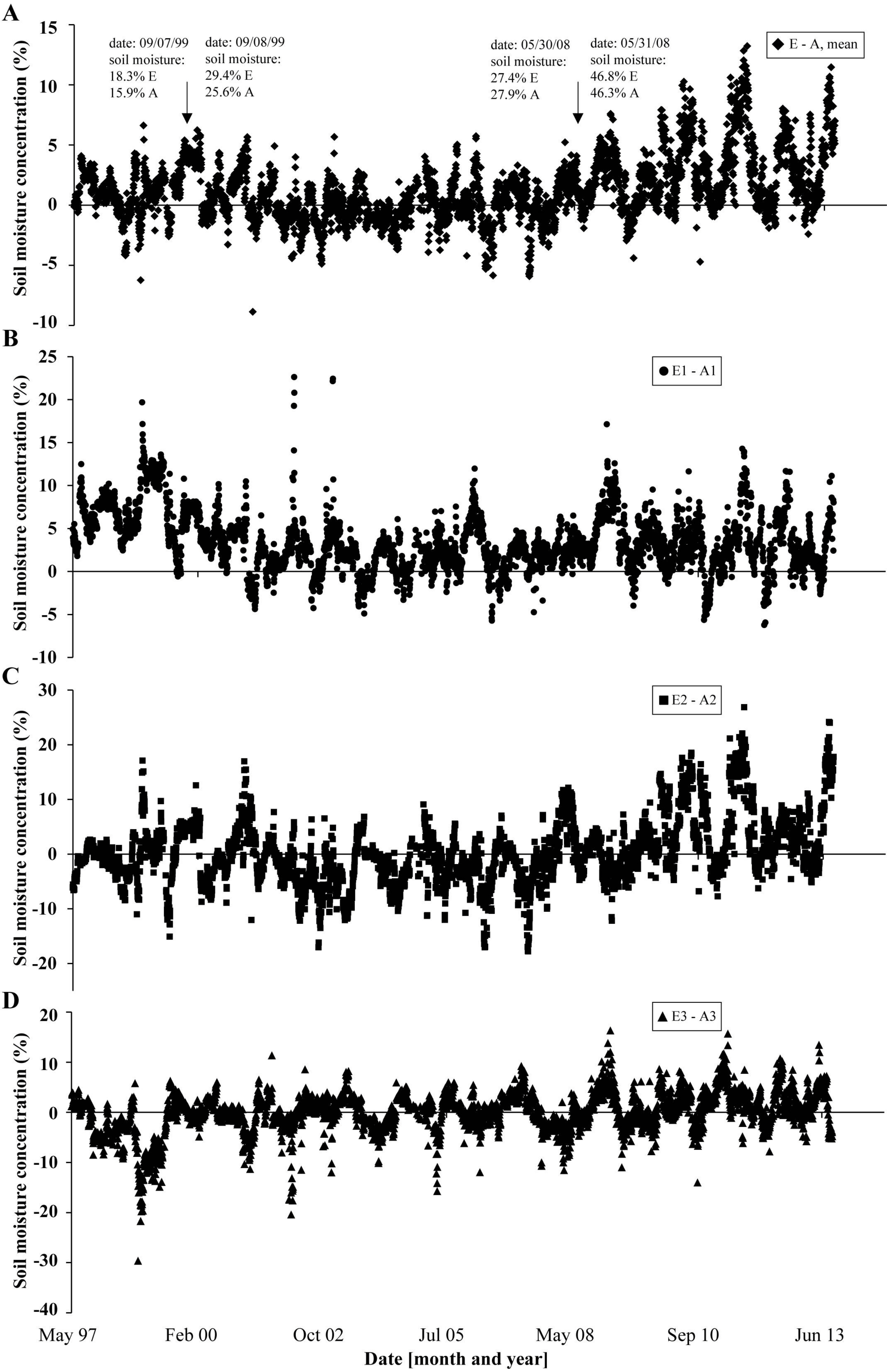

Figure S2. Differences in soil moisture concentration between eCO_2 and aCO_2 plots at GiFACE from 1997–2013: (A) Differences between mean soil moisture concentrations in elevated (E) and ambient (A) CO_2 plots. (B)–(D) Differences between soil moisture in the three sets (B, E1/A1; C,E2/A2; D, E3/A3).
