# Supplementary material for: Soil Conditions Rather Than Long-Term Exposure to Elevated CO2 Affect Soil Microbial Communities Associated with N-Cycling
Source: Front Microbiol. 2017 Oct 18;8:1976. doi: 10.3389/fmicb.2017.01976 (PMC5651278; doi:10.3389/fmicb.2017.01976)
Supplement: Supplementary file 8 [file Image3.pdf]

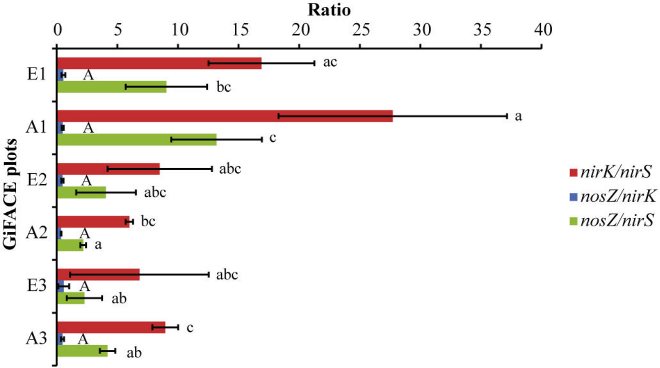

Figure S3. Ratios in copy numbers of denitrification genes *nirK*/*nirS*, *nosZ*/*nirS* and *nosZ*/*nirK* in soil of GiFACE plots. (Mean ± SD, n=3). Different letters indicate significant differences in the ratios for all plots.
